# Utilizing an Oxidized Biopolymer to Enhance the Bonding of Glass Ionomer Luting Cement Particles for Improved Physical and Mechanical Properties

**DOI:** 10.3390/biomimetics8040347

**Published:** 2023-08-05

**Authors:** Hanan Alsunbul, Aftab Ahmed Khan, Merry Angelyn Tan De Vera, Leonel S. J. Bautista, Ravish Javed

**Affiliations:** 1 Restorative Dentistry Department, College of Dentistry, King Saud University, Riyadh 11451, Saudi Arabia; halumbol@ksu.edu.sa; 2 Dental Health Department, College of Applied Medical Sciences, King Saud University, Riyadh 11451, Saudi Arabia; 3 Research Department, College of Dentistry, King Saud University, Riyadh 11451, Saudi Arabia; mtandevera@ksu.edu.sa; 4 Dental and Oral Rehabilitation Department, College of Applied Medical Sciences, King Saud University, Riyadh 11451, Saudi Arabia; lbautista@ksu.edu.sa; 5 Biomedical Technology Department, College of Applied Medical Sciences, King Saud University, Riyadh 11451, Saudi Arabia; rkhan1@ksu.edu.sa

**Keywords:** glass ionomer cement, gum arabic, luting cement, film thickness, mechanical properties, physical properties

## Abstract

This study aimed to determine the reinforcing effect of two weight ratios of Gum Arabic (GA) natural biopolymer, i.e., 0.5% and 1.0% in the powdered composition of glass ionomer luting cement. GA powder was oxidized and GA-reinforced GIC in 0.5 and 1.0 wt.% formulations were prepared in rectangular bars using two commercially available GIC luting materials (Medicem and Ketac Cem Radiopaque). The control groups of both materials were prepared as such. The effect of reinforcement was evaluated in terms of microhardness, flexural strength (FS), fracture toughness (FT), and tensile strength (TS). The internal porosity and water contact angle formation on the study samples were also evaluated. Film thickness was measured to gauge the effect of micron-sized GA powder in GA–GIC composite. Paired sample *t*-tests were conducted to analyze data for statistical significance (*p* < 0.05). The experimental groups of both materials containing 0.5 wt.% GA–GIC significantly improved FS, FT, and TS compared to their respective control groups. However, the microhardness significantly decreased in experimental groups of both cements compared to their respective control groups. The addition of GA powder did not cause a significant increase in film thickness and the water contact angle of both 0.5 and 1.0 wt.% GA–GIC formulations were less than 90^o^. Interestingly, the internal porosity of 0.5 wt.% GA–GIC formulations in both materials were observed less compared to their respective control groups. The significantly higher mechanical properties and low porosity in 0.5 wt.% GA–GIC formulations compared to their respective control group indicate that reinforcing GA powder with 0.5 wt.% in GIC might be promising in enhancing the mechanical properties of GIC luting materials.

## 1. Introduction

Indirect dental restorations, regardless of their fabrication method, require the use of a “luting agent” for proper sealing. Restorative dentistry has witnessed the utilization of various luting materials over time. Several important characteristics must be met to qualify as an ideal luting agent. Firstly, it should exhibit biocompatibility and possess caries and plaque prevention properties to promote oral health. Resistance to microleakage is another crucial attribute [1]. The luting agent should demonstrate sufficient strength to endure functional forces over the entire lifespan of the restoration. It should have low water solubility and minimal water sorption. in addition to being adhesive, radiopaque, esthetic, easy to manipulate, cost-effective, and have low viscosity at mixing [1,2].

Depending upon the longevity of the restoration, a luting agent can be provisional (short-term) or definitive (long-term) [3]. Provisional luting agents include zinc oxide-eugenol cement, non-eugenol-containing zinc oxide cement, and calcium hydroxide pastes. Conversely, definitive luting agents consist of zinc phosphate cement, zinc polycarboxylate cement, glass ionomer cement (GIC), resin-modified glass ionomer cement (RMGIC), and resin cement [1]. Among these materials, self-adhesive resin cement emerged in the dental market in 2002 [4]. In addition to the inherent limitations of resin cement, including its lack of anti-cariogenic properties, the application of RMGIC is characterized by its susceptibility to moisture contamination and the occurrence of polymerization shrinkage [5]. However, GIC stands out as a superior luting material due to its fluoride-releasing properties and ability to recharge fluoride from external sources [6]. This characteristic enables GIC to sustain a consistent level of fluoride, acting as a fluoride reservoir, and subsequently reducing demineralization [7].

Despite all the impressive properties that GIC holds, it has several drawbacks including sensitivity to a lack of moisture. Prolonged exposure to water can further exacerbate the issue, leading to heightened water sorption, increased plasticity, and hygroscopic expansion [8]. Historically, conventional GICs are mechanically weak and are not indicated for restoring stress-bearing areas, thus restricting their use [9,10]. Over time, several studies have compared the success of restorations cemented with different luting agents. Several studies, including those conducted by Yilmaz et al., have revealed lower retention and higher microleakage in crowns cemented with GIC compared to resin cement [11,12]. Another study highlighted the significantly superior retentive strength of resin cement compared to GIC [13]. Yet, in another study, the retentive strength of resin cement was observed better than RMGIC and conventional GIC [14].

Careful consideration of the mechanical weaknesses of GIC luting material has led us to postulate a study that investigates the potential benefits of incorporating Gum Arabic (GA) powder into commercially available GIC formulations. GA, a naturally derived biopolymer holds promise for enhancing the mechanical properties of GIC when introduced into its powdered composition [15]. Notably, GA possesses inherent antimicrobial activity and serves as a non-toxic natural excipient utilized for sustained drug release [16]. It is extracted from the hardened exudates of plants Acacia Senegal and Acacia Seyal [17]. We assume that the binding affinity between GA and the ceramic particles within the GIC powder could offer notable enhancements to both the FS and FT of the GIC luting material due to the strong properties that GA holds.

This study was directed towards modifying the powder composition of conventional GIC luting material by adding micro-sized oxidized GA powder to improve the mechanical properties which are necessary for clinical longevity. Specifically, the study aimed to investigate the influence of varying loading percentages of GA powder on the surface, physical, and mechanical properties of the luting GIC material. We hypothesized that the addition of GA powder to GIC would improve the tested properties.

## 2. Materials and Methods

### 2.1. Oxidization of GA Powder

The finest grade GA powder (Jacquard, Healdsburg, CA, USA) was obtained and ground further using mortal and pastel. One gram of GA powder was combined with 20 mL of distilled water. The mixture was heated to 70 degrees Celsius and stirred for 30 min. Following that, 30 mL of 30% H_2_O_2_ was gradually added along with a catalytic quantity of ferrous sulfate. The reaction mixture was further heated to 100 degrees Celsius and maintained at that temperature for 2 h. Throughout this period, distilled water was added incrementally to ensure that the overall volume of the mixture remained constant. Once the reaction was confirmed to be completed through a peroxide strip test, the water was evaporated under vacuum conditions.

### 2.2. Sample Preparation

In this study, two commercially available Type 1 GIC, i.e., Medicem (Promedica, Dental Material GmbH, Neumuenste, Germany) and Ketac Cem Radiopaque (3M ESPE, Seefeld/Oberbay, Germany) were selected and supplied as a powder–liquid system. To enhance their properties, oxidized GA powder with a particle size ranging from 50 to 100 microns was incorporated into the powder composition of GIC at two different weight percentages (0.5% and 1.0%). The mixture consisting of the GIC and Gum Arabic powder was manually mixed. Subsequently, to ensure optimal mixing, the mixture was placed on a vibrator for 5 min. Control groups for each brand of GIC were prepared with 0 wt.% GA. Both the control and experimental GIC samples were mixed with the liquid component of the cement following the recommended powder-to-liquid ratio, specifically 1:1.

To fabricate the samples, a rectangular silicon mold with dimensions of 25 mm × 2 mm × 2 mm was utilized. The powder and liquid components of each study group were mixed using a plastic spatula until a paste-like consistency was attained. The mixing process was conducted for no more than 1 min. This paste was then poured into the silicon mold, filling it up to the brim. The working time ranged between 1 to 2 min. After 30 min, the samples were carefully removed from the mold and placed into labeled containers. These containers were stored in an incubator at a temperature of 37 °C with 100% humidity for 7 days. It is important to note that all samples were prepared by a single trained operator under controlled room temperature conditions of 21 °C.

### 2.3. Micro-Computed Tomography (Micro CT) Test

To investigate the three-dimensional architecture of the experimental samples, a micro-computed tomography system (Skyscan 1172, Bruker, Aartselaar, Belgium) was employed. A single sample was randomly selected from each study group to evaluate the porosity %. The scanning parameters included a voltage of 100 kV, a current of 50 μA, and voxel dimensions of 14.2 μm. This allowed for the evaluation of potential pores in the cylindrical-shaped samples. The scanning procedure involved a complete 360° rotation around the vertical axis [18]. Subsequently, the proprietary software’s porosity tool was utilized to compute the total porosity values.

### 2.4. Microhardness Test

The microhardness evaluation of each group (*n* = 10) was conducted using a Vickers Microhardness Tester (Model 402 MVD, ITW Test and Measurement Co., Ltd., Shanghai, China). The assessment employed a Vickers diamond indenter with a magnification of 20×. Microhardness measurements were obtained by applying an indentation to the central region of the sample, resulting in Vickers hardness numbers (VHN). The measurement procedure involved applying a 25-g load for 15 s.

### 2.5. Flexural Strength (FS) Test

To determine the FS, a 3-point bending test was performed on test samples from each study group, with a total of 10 samples (*n* = 10). The 3-point bending test was conducted following the ISO 9917-2:2017 standard. For the testing procedure, all specimens were placed on a universal testing machine (Model no. 3369 Instron, Canton, MA, USA). A load cell with a capacity of 30 kN was used and the cross-head speed was set at 0.5 mm/min. During the test, the maximum load at fracture and the FS (in megapascal, MPa) were automatically recorded using the proprietary software (Bluehill ver. 2.4) associated with the testing machine.

### 2.6. Fracture Toughness (K_IC_) Test

A 3-point bending test was utilized, employing a universal testing machine to evaluate the FT values of each group under investigation. The beam-shaped sample with dimensions of 25 mm × 2 mm × 2 mm was selected and a notch with 0.5 mm width and 2.0 mm depth was prepared. Each sample was carefully positioned within the apparatus, utilizing a custom-designed supporting jig to stabilize the beam at its ends. To induce fracture, a chisel-shaped blade uniformly applied the force until the fracture was realized. The universal testing machine was operated at a controlled crosshead speed of 0.5 mm/min. The determination of FT involved the calculation of the critical stress intensity factor (K_IC_), as indicated by the following equation:

K_IC_ = g [(P_max_ {S_o_10 ^−6^}/(BW ^3/2^)] [(3 (a/W) ^1/2^)/(2 (1 − a/W) ^3/2^)] (1)

g = g(a/W) = 1.99 − [a/W] [1 − a/W] [2.15 − 3.93 (a/W) + 2.7 (a/W) ^2^]/(1 + 2 (a/W)) (2)

where,

K_IC_ = Stress intensity factor.

g = A dimensionless function that depends on the geometry of the crack and the material properties.

P_max_ = The maximum applied load.

S_o_ = Initial crack length.

BW = Width of the sample.

a = Crack length.

W = Length of the crack in the sample.

### 2.7. Tensile Strength (TS) Test

The TS of the study samples was assessed utilizing a universal testing machine with a test conducted at a cross-head speed of 0.5 mm/min and a load cell set at 30 kN. Each group included a bar-shaped specimen (*n* = 10) with the dimensions of 25 mm × 2 mm × 2 mm that was affixed to the grips of a tensile device using cyanoacrylate (Super Glue, Henkel/Loctite, Westlake, CA, USA). The proprietary software was employed to record the failure loads in newtons (N) and the TS in megapascals (MPa).

### 2.8. Film Thickness Test

To determine the film thickness, the dispensing and mixing method for the control and experimental groups was according to the manufacturer’s direction. The thickness of two glass plates was accurately measured to 0.01 μm and referred to as measurement (A). A mixture of each cement, with a volume of 0.1 ± 0.05 mL, was placed in the center of one glass plate. Subsequently, the second glass plate was placed on top, covering the mixture. A vertical load of 150 N using a universal testing machine was applied to the center of the specimen for 10 s. After a lapse of ten minutes from the application of the load, the thickness of the two plates was measured again. This measurement was denoted as measurement (B). The difference between the two measurements (B − A) represented the film thickness. This process was repeated five times to determine the average film thickness for each study group.

### 2.9. Water Contact Angle Test

Contact angle measurement was performed using a Contact Angle Tensiometer (Theta Lite, Dyne Technology, Staffordshire, UK) to assess the variations in contact angle resulting from the varying weight ratios of GA powder in the composition of Glass Ionomer Cement (GIC). The contact angle of water was determined by measuring the angle formed by a water droplet placed on the surface of the sample after an elapsed time of 20 s.

### 2.10. Statistical Analysis

To analyze the effects of different weight ratios of GA in GIC, a paired sample *t*-test was conducted to evaluate the mean differences between the control and experimental groups. A *p*-value of exactly 0.05 or less was considered significant. All statistical calculations were carried out using SPSS 28.0 for Windows (SPSS Corporation, Chicago, IL, USA).

## 3. Results

**Figure 1:**
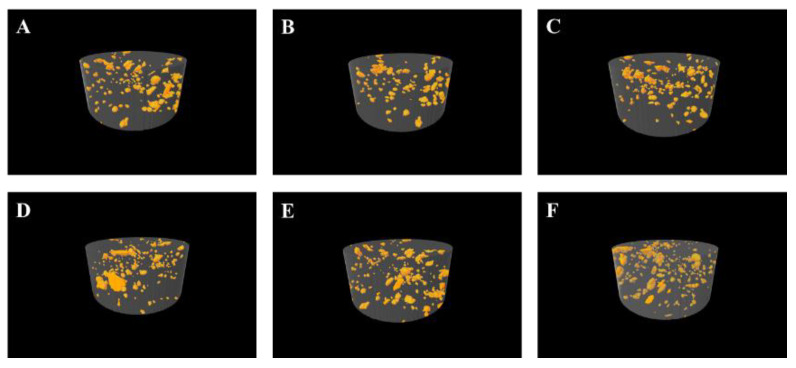
Three-dimensional images of micro CT for porosity evaluation: (**A**–**C**) depicts the internal porosity of the control, 0.5 wt.% GA–GIC, and 1.0 wt.% GA–GIC with Medicem, respectively; (**D**–**F**) depicts the internal porosity of the control, 0.5 wt.% GA–GIC and 1.0 wt.% GA–GIC with Ketac Cem Radiopaque.

Table 1 presents the porosity % of the study samples. The 1.0 wt.% GA–GIC composite in both materials exhibited higher total porosity % compared to their respective control groups. Surprisingly, 0.5 wt.% formulations of GA–GIC composite in both materials demonstrated a lower total porosity % (23.84% and 26.18% in Medicem and Ketac Cem Radiopaque, respectively) compared to their respective control groups. The pictorial representations of the study groups are demonstrated in Figure 1.

**Figure 2:**
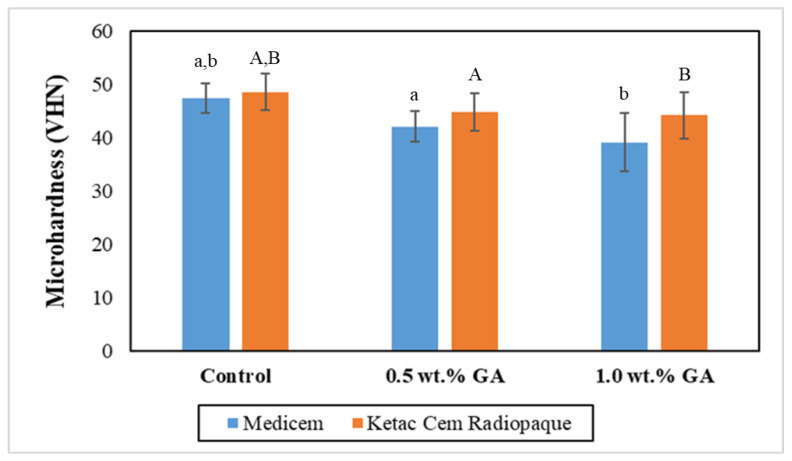
Bar graph representing the mean microhardness with the error bars of the control and experimental groups. Key: Same lower-case alphabets show significant differences within the groups of Medicem while the same upper-case alphabets show significant differences within the groups of Ketac Cem Radiopaque.

Figure 2 graphically illustrates the mean microhardness values of the study groups. The results of the independent two-tailed *t*-test revealed significant differences between the control group and the representative experimental groups for both Medicem GIC and Ketac Cem Radiopaque. However, statistically insignificant differences were observed among the experimental groups of both Medicem and Ketac Cem Radiopaque. Notably, the control group of Ketac Cem Radiopaque exhibited the highest mean microhardness (48.63 ± 3.46 VHN) while the lowest microhardness was observed in the Medicem group with 1.0 wt.% GA (39.19 ± 5.41 VHN).

**Figure 3:**
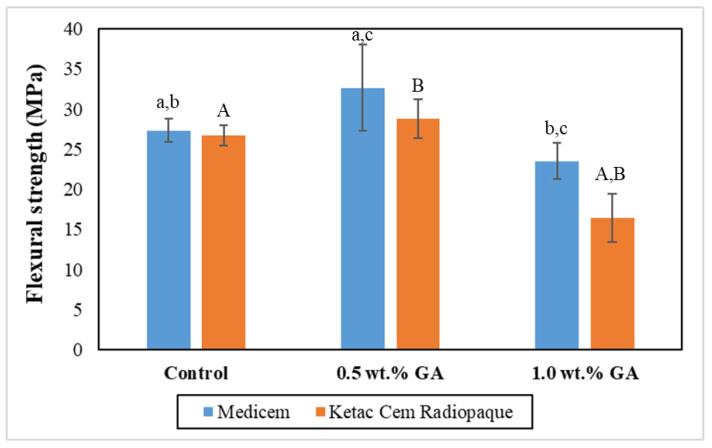
Bar graph representing the mean FS with the error bars of the control and experimental groups. Key: Please Same lower-case alphabets show significant differences within the groups of Medicem while the same upper-case alphabets show significant differences within the groups of Ketac Cem Radiopaque.

Figure 3 graphically illustrates the mean FS values of the study groups. The results of the independent two-tailed *t*-test revealed significantly higher values of 0.5 wt.% GA in the group of Medicem compared to the control group and 1.0 wt.% GA group of Medicem. In contrast, 0.5 wt.% GA group of Ketac Cem Radiopaque showed insignificantly higher values compared to the control and significantly higher values compared to the 1.0 wt.% GA group of Ketac Cem Radiopaque. Notably, the 0.5 wt.% GA group of Medicme exhibited the highest mean FS (32.66 ± 5.37 MPa) while the lowest FS was observed in the Ketac Cem Radiopaque group with 1.0 wt.% GA (16.44 ± 2.96 MPa).

**Figure 4:**
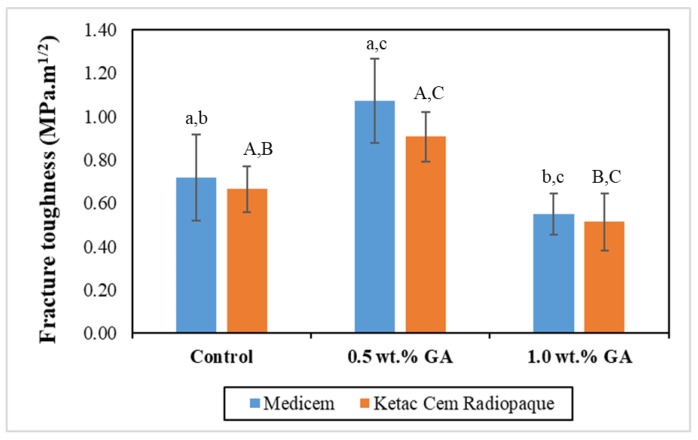
Bar graph representing the mean FT with the error bars of the control and experimental groups. Key: Same lower-case alphabets show significant differences within the groups of Medicem while the same upper-case alphabets show significant differences within the groups of Ketac Cem Radiopaque.

Figure 4 graphically illustrates the mean FT values of the study groups. The results of the independent two-tailed *t*-test revealed a significantly higher value of 0.5 wt.% GA groups of both Medicem and Ketac Cem Radiopaque GICs compared to their respective control and 1.0 wt.% GA groups. However, both control groups exhibited significantly higher FT values compared to their respective 1.0 wt.% GA groups.

**Figure 5:**
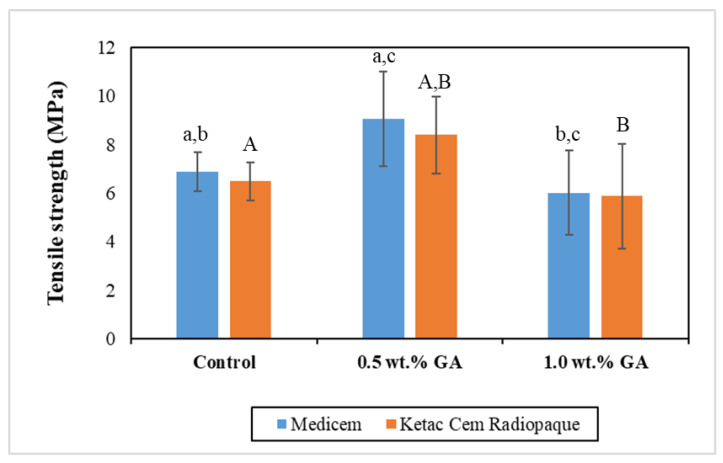
Bar graph representing the mean TS with the error bars of the control and experimental groups. Key: Same lower-case alphabets show significant differences within the groups of Medicem while the same upper-case alphabets show significant differences within the groups of Ketac Cem Radiopaque.

Figure 5 graphically presents the mean TS values of the various study groups. Statistical analysis using an independent two-tailed *t*-test revealed a noteworthy increase in the 0.5 wt.% GA group of both Medicem and Ketac Cem Radiopaque GICs as compared to their respective control and 1.0 wt.% GA groups. Specifically, the control group of Medicem GIC demonstrated significantly higher FT values in contrast to its corresponding 1.0 wt.% GA groups. Conversely, the control group of Ketac Cem Radiopaque exhibited a comparatively higher value, albeit statistically insignificant, when compared to its corresponding 1.0 wt.% GA group.

Table 2 presents the film thickness measurements for the control and experimental groups. The control groups for both GICs demonstrated lower film thickness values in comparison to their respective experimental groups. However, the film thickness values of the experimental groups were found to be insignificantly higher compared to their respective control groups. Among all the study groups, the G1 group of Ketac Cem Radiopaque exhibited the lowest mean film thickness value (15 ± 4 µm) while the G3 group of Medicem displayed the highest mean film thickness value (24 ± 5 µm).

The average water contact angles observed in all experimental groups exhibited significantly higher values compared to their respective control groups (*p* < 0.05). The inclusion of GA powder in the composition of Glass Ionomer Cement (GIC) had an adverse impact on the water contact angle. Among the experimental groups, the highest contact angle was recorded in the presence of 1.0 wt.% GA in Medicem GIC (86.82 ± 3.13°) while the control group utilizing Ketac Cem Radiopaque GIC displayed the lowest contact angle (65.51 ± 1.85°). The details are in Table 3.

## 4. Discussion

In our previous study, GA was successfully oxidized and incorporated into GICs at weight ratios of 0.5 and 1.0 wt.%. We observed that oxidation resulted in the formation of various acid groups, including but not limited to glucuronic acid, galacturonic acid, glucaric acid, and guluronic acid, both in small and large molecular structures and resulted in improved mechanical properties of the GIC luting material, including nano hardness, elastic modulus, compressive strength, and diametral tensile strength [19]. The observed enhancements in compressive strength, diametral tensile strength, and elastic modulus of 0.5 and 1.0 wt.% GA–GIC formulations provided substantial motivation to persist with further investigation in this research endeavor. Therefore, in continuation of the previous work, this study envisaged evaluating other important physical and mechanical properties of the GA–GIC composite luting materials. The findings of this study revealed a significant improvement in the mechanical properties using 0.5 wt.% GA in GIC compared to the control. However, the microhardness was significantly reduced using the experimental formulations. Consequently, the hypothesis posited in this study is partially accepted. All the tested properties significantly improved with 0.5 wt.% GA–GIC formulation except for microhardness.

The reinforcement of GIC luting with GA powder caused a reduction in microhardness. This is because natural biopolymers typically possess lower stiffness and hardness [20] compared to inorganic components found in GIC. The flexible and deformable nature of rubbery material can reduce the overall rigidity and hardness of the set cement [21]. GA particles may hinder the packing of inorganic particles, leading to a more compliant and softer material. The extent of microhardness reduction in GIC due to GA can vary depending on factors such as the type, concentration, and compatibility of the polymer with the cement matrix.

The modified GICs with 0.5 wt.% GA significantly enhanced the FS of both luting cements. The reaction between a biopolymer and a weak acid in GIC liquid composition may result in some level of chemical breakdown of the biopolymer which could cause it to soften or dissolve to some extent leading to better particle packing between the GICs glass particles. The dissolved GA may act as additional bonding sites for the polyacrylic polymer which results in strengthening GICs [22]. This improved interfacial bonding enhances the load transfer ability within the material, resulting in increased FS.

The enhanced FT of 0.5 wt.% GA–GIC composite in both cements could be attributed to the reduced brittleness. GICs typically exhibit some degree of brittleness which can limit their mechanical properties. When a natural biopolymer is incorporated, it can help improve the toughness and ductility of the material. This reduction in brittleness allows the GIC to withstand higher flexural stresses without fracturing [23]. Additionally, 0.5 wt.% GA may homogeneously incorporate in the glass component and disperse the applied stress more evenly throughout the material, absorbing and distributing the stress more effectively. This dispersion reduces the concentration of stress at localized areas, preventing the propagation of cracks and improving the FT [24] of the GIC. Reduced FT in 1.0 wt.% GA formulations could be due to the overcrowding of filler particles which may interfere with the polysalt bridge formation within the GIC [25].

Due to its limited clinical utility attributed to a weak TS [26], GIC luting poses certain constraints. Again, a significantly higher TS using 0.5 wt.% GA–GIC composite in both cements could be attributed to the ability of GA biopolymer to fill in the micropores or voids within the GIC structure which would otherwise act as stress concentration points. By occupying these spaces, the biopolymers help distribute the applied stress more evenly, thereby reducing the likelihood of crack initiation and propagation. The hydrophilic nature of GA may impede the premature escape of water, thereby allowing it to be firmly bound through the hydration of cations released from the glass or siloxane groups present on the surface of glass particles [27,28]. The early loss of water diminishes the degree of cross-linking and elevates the porosity of the cement [23], consequently resulting in a weakened TS. Additionally, GA encompasses water-soluble polysaccharides capable of forming hydrogen bonds with the polyacrylic acid component of the cement. These hydrogen bonds contribute to the creation of a more robust and cohesive matrix within the cement, thereby yielding a material that exhibits heightened resistance to tension.

It is notable to mention that lower weight ratios of GA did not affect the film thickness of GA–GIC composite luting cement. This might hint toward non-agglomerated film formation leading to the homogenous dispersion of GA within the GIC mixture. The polyacrylic acid of the liquid composition of the GIC might have dissolved the GA powder and filled the internal voids during the setting reaction of the GIC. Polyacrylic acid may act as a solubilizing agent or a carrier for the biopolymer, enhancing its dispersibility in a solution. This proposition was further supported by the micro CT measurements when it was observed that the total % of voids was slightly reduced in 0.5 wt.% GA–GIC composite compared to the control groups in both cements. Contrary to our initial postulation that the inclusion of GA powder would result in a reduction in the water contact angle due to the hydrophilic properties of GA, our observations demonstrated the opposite effect in GA–GIC composites. However, the increase in the water contact angle was insignificant in both 0.5 and 1.0 wt.% GA–GIC formulations.

In contrast to the positive results observed with 0.5 wt.% of GA in GIC formulation, the detrimental impact of a 1.0 wt.% GA in GIC might suggest that an increased GA weight ratio may hinder the formation of effective interfacial bonding between the glass particles and the GIC matrix. High weight ratios of GA can contribute to an increase in the porosity of the GIC matrix as observed in micro CT evaluation. The presence of excessive voids and gaps within the material can act as stress concentration points and weaken the overall strength [29]. GA, being a hydrophilic substance [15], has the potential to impede the hydration process of the cement. In GICs, the water content is crucial for proper setting and cross-linking reactions [30]. Excessive GA may hinder the access of water to reactive sites, resulting in incomplete hydration and reduced cross-linking density. The excessive presence of GA could disrupt the homogeneity and integrity of the glass particles, leading to decreased intermolecular interactions and weaker matrix cohesion. These factors may lead to weak load transfer and reduced flexion, tension, and FT.

The favorable outcomes of this laboratory study suggest that using GA–GIC-based luting cement for prosthetic restorations would prove beneficial for the long-term clinical survival of the dental prosthesis compared to resin-based luting cement. Additionally, the GIC chemical adheres to the tooth structure and helps to seal the restoration margins, reducing the risk of microleakage and secondary decay. The coefficient of thermal expansion of GICs is similar to that of tooth structure. This means that they expand and contract at a similar rate when exposed to temperature changes, minimizing the risk of debonding or microfractures at the restoration–tooth interface. The ability to release fluoride over time provides a potential benefit in terms of caries prevention and remineralization.

The oxidized biopolymers such as GA may exhibit synergistic effects when combined with conventional GICs. This means that their presence can interact with the existing components of the cement, such as the polyacid and glass particles, leading to improved properties. Peroxide-mediated oxidation of GA converts polysaccharides into reactive oxygen species, forming acid groups that modify the chemical structure [31]. The acid groups bind with silica, alumina, and calcium in GIC powder, lowering the pH and improving the reaction setting and strength. This synergy can result in increased mechanical properties as we observed in this experimental study. However, in this study, artificial aging was not considered to predict the long-term clinical performance of the GA–GIC composite. Additionally, shear bond strength evaluation of the GA–GIC composite with tooth substrate as well as with crown and bridge metallic framework is equally important to evaluate.

## 5. Conclusions

In conclusion, our research findings demonstrate that the incorporation of 0.5 wt.% GA micron-sized powder in the experimental GIC formulation led to significant improvements in FS, FT, and TS compared to the control group. However, it was observed that the microhardness of the 0.5 wt.% GA–GIC formulation decreased compared to the control group. In contrast, the inclusion of 1.0 wt.% GA in the GIC formulation resulted in a deterioration of the tested properties. These results suggest that GA, when added at an optimal concentration, can enhance the mechanical properties of GIC. Further investigations are warranted to explore the underlying mechanisms and to optimize the GA content for improved performance in GIC formulations.

## Figures and Tables

**Table 1:** Micro CT of study samples for total porosity (in %) evaluation.

Material	Group	Closed Porosity (%)	Open Porosity (%)	Total Porosity (%)
Medicem	Control	0.06	24.87	24.92
	0.5 wt.% GA–GIC	0.35	23.57	23.84
	1.0 wt.% GA–GIC	0.68	24.61	25.13
Ketac Cem Radiopaque	Control	0.28	26.50	26.70
	0.5 wt.% GA–GIC	0.62	25.73	26.18
	1.0 wt.% GA–GIC	0.98	28.53	29.23

**Table 2:** Film thickness mean and standard deviation (SD) of the control and experimental groups.

Group	Film Thickness (µm)	
	Medicem	Ketac Cem Radiopaque
G1 (Control)	21 ± 3	15 ± 4
G2	23 ± 3	17 ± 4
G3	24 ± 5	19 ± 3

Insignificant differences within the material groups.

**Table 3:** Tabulation of mean water contact angle with the error bars of the control and experimental groups.

Group	Mean Water Contact Angle (°)	Image
Medicem (control)	72.55 ± 3.51	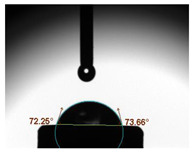
Medicem 0.5 wt.% GA	82.46 ± 2.97	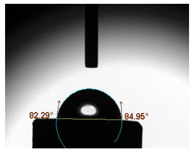
Medicem 1.0 wt.% GA	86.82 ± 3.13	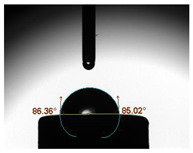
Ketac (control)	65.51 ± 1.85	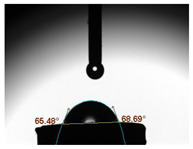
Ketac Cem Radiopaque 0.5 wt.% GA	74.81 ± 2.37	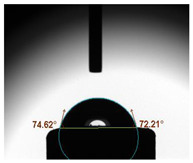
Ketac Cem Radiopaque 1.0 wt.% GA	83.16 ± 2.89	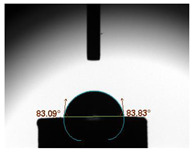

## Data Availability

The data presented in this study are available on request from the corresponding author.
